# MicroRNA-155 attenuates late sepsis-induced cardiac dysfunction through JNK and β-arrestin 2

**DOI:** 10.18632/oncotarget.17636

**Published:** 2017-05-04

**Authors:** Yu Zhou, Yan Song, Zahir Shaikh, Hui Li, Haiju Zhang, Yi Caudle, Shouhua Zheng, Hui Yan, Dan Hu, Charles Stuart, Deling Yin

**Affiliations:** ^1^ Department of Internal Medicine, College of Medicine, East Tennessee State University, Johnson City, TN 37614, USA; ^2^ Department of Neurology, Renmin Hospital of Wuhan University, Wuhan 430060, China; ^3^ Department of Vascular Surgery, The First Affiliated Hospital of Zhengzhou University, Zhengzhou 450052, China; ^4^ Department of Thyroid Surgery, The First Affiliated Hospital of Zhengzhou University, Zhengzhou 450052, China

**Keywords:** microRNA-155, late sepsis, cardiac dysfunction, β-arrestin 2, inflammatory

## Abstract

Cardiac dysfunction is correlated with detrimental prognosis of sepsis and contributes to a high risk of mortality. After an initial hyperinflammatory reaction, most patients enter a protracted state of immunosuppression (late sepsis) that alters both innate and adaptive immunity. The changes of cardiac function in late sepsis are not yet known. MicroRNA-155 (miR-155) is previously found to play important roles in both regulations of immune activation and cardiac function. In this study, C57BL/6 mice were operated to develop into early and late sepsis phases, and miR-155 mimic was injected through the tail vein 48 h after cecal ligation and puncture (CLP). The effect of miR-155 on CLP-induced cardiac dysfunction was explored in late sepsis. We found that increased expression of miR-155 in the myocardium protected against cardiac dysfunction in late sepsis evidenced by attenuating sepsis-reduced cardiac output and enhancing left ventricular systolic function. We also observed that miR-155 markedly reduced the infiltration of macrophages and neutrophils into the myocardium and attenuated the inflammatory response via suppression of JNK signaling pathway. Moreover, overexpression of β-arrestin 2 (Arrb2) exacerbated the mice mortality and immunosuppression in late sepsis. Furthermore, transfection of miR-155 mimic reduced Arrb2 expression, and then restored immunocompetence and improved survival in late septic mice. We conclude that increased miR-155 expression through systemic administration of miR-155 mimic attenuates cardiac dysfunction and improves late sepsis survival by targeting JNK associated inflammatory signaling and Arrb2 mediated immunosuppression.

## INTRODUCTION

Sepsis is defined as a systemic inflammatory response to life-threatening infection with the presence of organ failure [1]. Despite progress in antibiotic treatment and prompt institution of life support, sepsis remains the major cause of death in intensive care units [2]. The mechanism involved in the host immune response to sepsis is debated. Traditionally, the immune response was considered to be typified by a hyperinflammatory status with excessive production of pro-inflammatory mediators by macrophages and neutrophils [3, 4]. Until recently, most research was focused on ameliorating the hyperinflammatory response of the disorder, however, clinical trials using anti-inflammatory treatment failed to reduce overall sepsis mortality [5]. In a postmortem study of sepsis, more than 70% of patients died after an early/acute hyperinflammatory phase and most patients enter a state of late/chronic hypoinflammation and immunosuppression [6, 7]. So far there is no effective therapy for late sepsis.

Growing evidence suggests that cardiovascular dysfunction as a major complication of sepsis is correlated with worse performance and poor prognosis, and cardiac dysfunction increases the risk of mortality rates up to 70% in sepsis/septic shock [8, 9]. Therefore, it is a critically unmet need to clarify the mechanisms of cardiac dysfunction caused by sepsis and explore effective treatment to ameliorate the sepsis prognosis.

Over the past few years, microRNAs (miRs) have proved to be critical regulators of physiological homeostasis in the cardiovascular system [10, 11]. A subset of miRs has been identified as potential prognostic indicators of heart failure following acute myocardial infarction (MI). Using a high-throughput array method, serum expression of miR-155 and miR-380 in patients who experienced cardiac death were approximately 3-4 fold higher compared with those who returned for their 1-year follow-up [12]. It has been shown that miR-155 exerts an antiangiogenic but proarteriogenic role in the regulation of neovascularization, and miR-155 down- regulation would result in impaired arteriogenesis after MI and could exacerbate further injury to the myocardium [13]. However, the role of miR-155 in the progression of cardiac dysfunction in sepsis has not yet been elucidated.

β-arrestin 2 (Arrb2), a multifunctional regulator of G protein-coupled receptors with especially high expression in cardiovascular tissues, can modulate a diverse array of biological processes [14, 15]. Increasing evidence suggests that Arrb2 plays a crucial role in regulating systemic immune responses and the kinetics of Arrb2-mediated signaling is tissue-dependent [15, 16]. Our previous studies found that miR-155 could directly target the 3′-UTR of Arrb2 mRNA in a sequence-specific manner and there is a loop pathway between Arrb2 and miR-155 to promote the transition of cardiac stem cells into cardiomyocytes [17]. However, the effect of Arrb2 on cardiac function during late sepsis remains unknown.

In the present study, we demonstrated that increased expression of miR-155 protects against cardiac dysfunction in late sepsis. We also observed that miR-155 markedly reduces the infiltration of macrophages and neutrophils into the myocardium and attenuates the inflammatory response via suppression of JNK activity. Overexpression of Arrb2 exacerbates the mortality and immunosuppression in late sepsis mice. Transfection of miR-155 mimic reduces Arrb2 expression in the myocardium, and restores mice immunocompetence and improves survival. These findings suggest that modulation of the miR-155/Arrb2 pathway is capable of preventing late sepsis-induced cardiac dysfunction.

## RESULTS

### *In vivo* transfection of miR-155 mimic improves late sepsis survival

**Figure 1:**
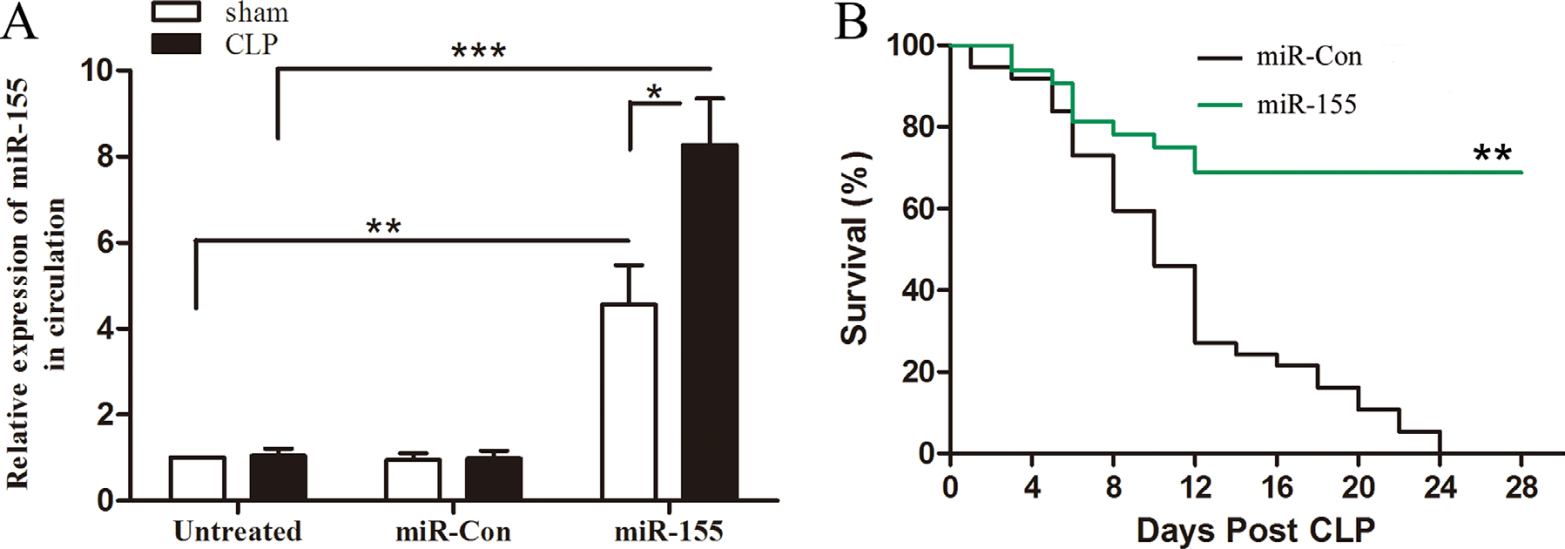
Increased expression of miR-155 in circulation improves late-septic mice survival outcome Eight- to 10-weeks C57BL/6 male mice were subjected to CLP, and sham surgical operation served as sham control. miR-155 mimic or miR-Con (80 mg/kg) was injected through the tail vein 48 h after CLP. (**A**) After 12 days of CLP, sera were harvested and the expression of miR-155 was determined by real time PCR. There were four mice per group. *p < 0.05, **p < 0.01, ***p < 0.001 compared with indicated groups. (**B**) After 48 h of CLP, mice were transfected with miR-155 mimic or miR-Con and then monitored for survival for up to 28 days. There were 15-18/group. **p < 0.01 compared with miR-Con group.

Previous study shows that miR-155 was increased in early sepsis and returned to baseline level in late sepsis [18]. To determine whether increased expression of miR-155 in late phase could impact the sepsis outcome, miR-155 mimic was injected through the tail vein 48 h after CLP to allow the initiation of sepsis and mortality was monitored for 28 days. As shown in Figure 1A, circulating miR-155 maintain baseline level on day 12 after CLP sepsis, and miR-155 mimic transfection significantly increased circulating miR-155 expression. The Kaplan-Meier survival curve (Figure 1B) indicated that 50% of the miR-Con treated septic mice died within 10 days, and 100% mortality occurred at 24 days after CLP. However, survival in miR-155 mimic treated septic mice was improved by 67% compared with miR-Con group. Our CLP model is operated to develop into early and late sepsis phases, which produces 20-30% mortality during early sepsis (days 1 to 5). We didn't observe the effect of miR-155 mimic on mortality until day 6 after CLP. These results suggest that increased miR-155 expression during late phase prevents sepsis mortality. In the following experiments, we intended to identify the underlying mechanism for the protective effect of miR-155 in late sepsis.

### Increased myocardium expression of miR-155 attenuates cardiac dysfunction in late sepsis

**Figure 2:**
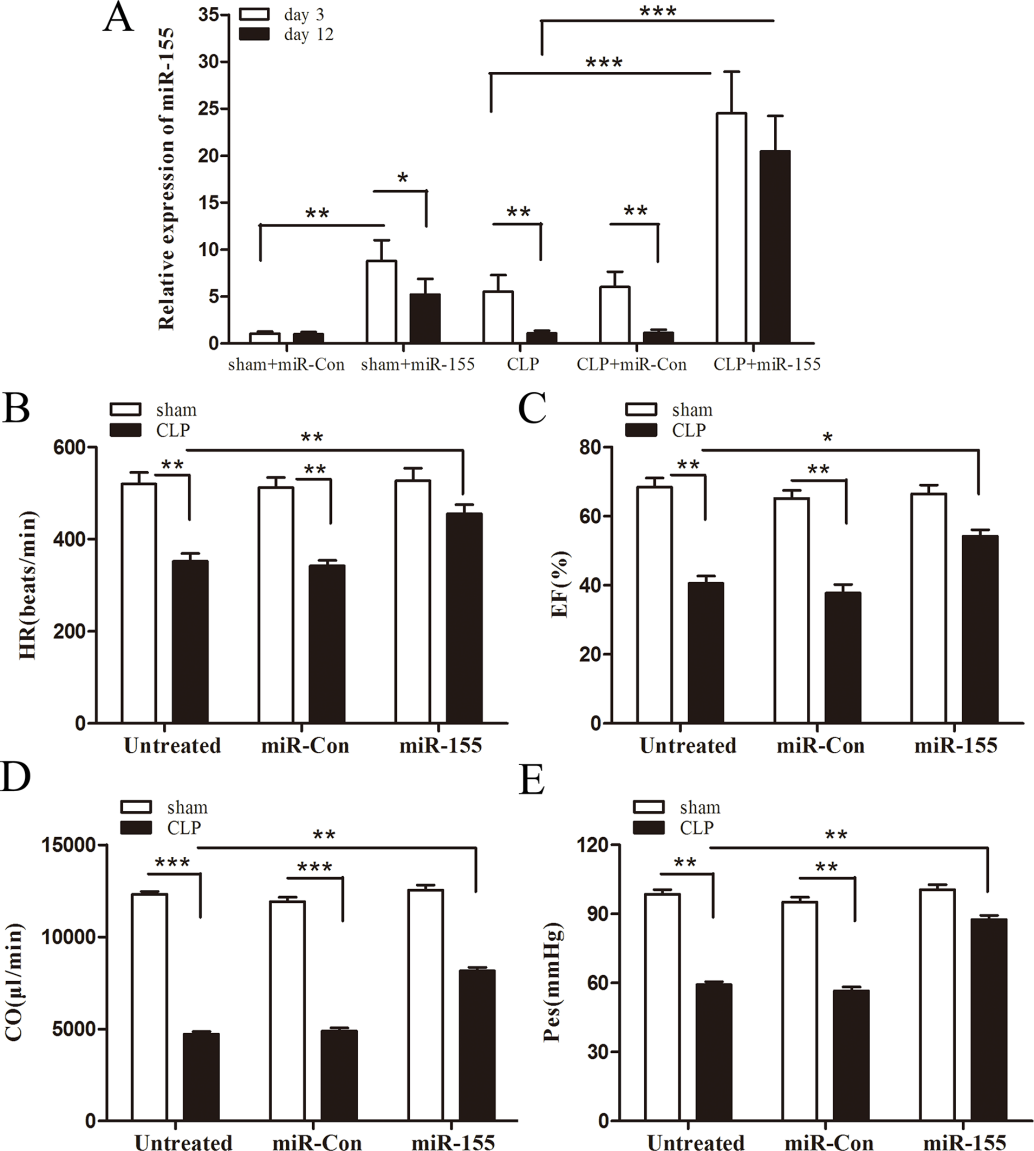
Increased expression of miR-155 in the myocardium attenuates cardiac dysfunction in late sepsis Eight- to 10-weeks old C57BL/6 male mice were subjected to CLP or sham surgical operation. miR-155 mimic or miR-Con (80 mg/kg) was injected through the tail vein 48 h after CLP. (**A**) After 3 days or 12 days of CLP, hearts were harvested and the level of miR-155 was determined by real time PCR. There were four mice per group. After 12 days of CLP, cardiac function measurement was analysis by pressure-volume loop hemodynamic parameters. (**B**) HR, heart rate. (**C**) EF, ejection fraction. (**D**) CO, cardiac output. (**E**) Pes, left ventricular end-systolic pressure. There were five mice per group. *p < 0.05, **p < 0.01, ***p < 0.001 compared with indicated groups.

Recently, it has been shown that cardiac dysfunction plays a pivotal role in sepsis- induced mortality [8, 9]. To determine the role of miR-155 in cardiac function during late sepsis, we firstly measured its level in the myocardium of septic mice. As shown in Figure 2A, the miR-155 level was increased approximately 5-fold in early sepsis (compared to sham control, set at 1-fold), and returned to baseline level in late sepsis (day 12). After transfection with miR-155 mimic, the expression of miR-155 was increased 24.5-fold compared with sham control on day 3, and sustained increase around 20-fold on day 12. To evaluate the effect of miR-155 on cardiac function in late sepsis, we collected hemodynamic parameters by pressure-volume loop measurement on day 12 after sepsis. CLP induced significant cardiac dysfunction in late sepsis mice as evidenced by decreased heart rate (32.3%) (Figure 2B), EF% (40.6%) (Figure 2C), cardiac output (61.7%) (Figure 2D), and Pes (39.9%) (Figure 2E) compared with baseline values. In contrast, miR-155 mimic treatment significantly attenuated CLP-induced cardiac dysfunction, which increased heart rate by 29.3%, EF% by 33.5%, cardiac output by 72.8%, and Pes by 47.8% when compared with the untreated CLP group. Taken together, increased expression of miR-155 in the myocardium attenuates sepsis-reduced cardiac output and enhances left ventricular systolic function, which may improve late sepsis mice survival.

### Transfection of miR-155 mimic attenuates infiltration of macrophages and neutrophils into the myocardium in late sepsis

**Figure 3:**
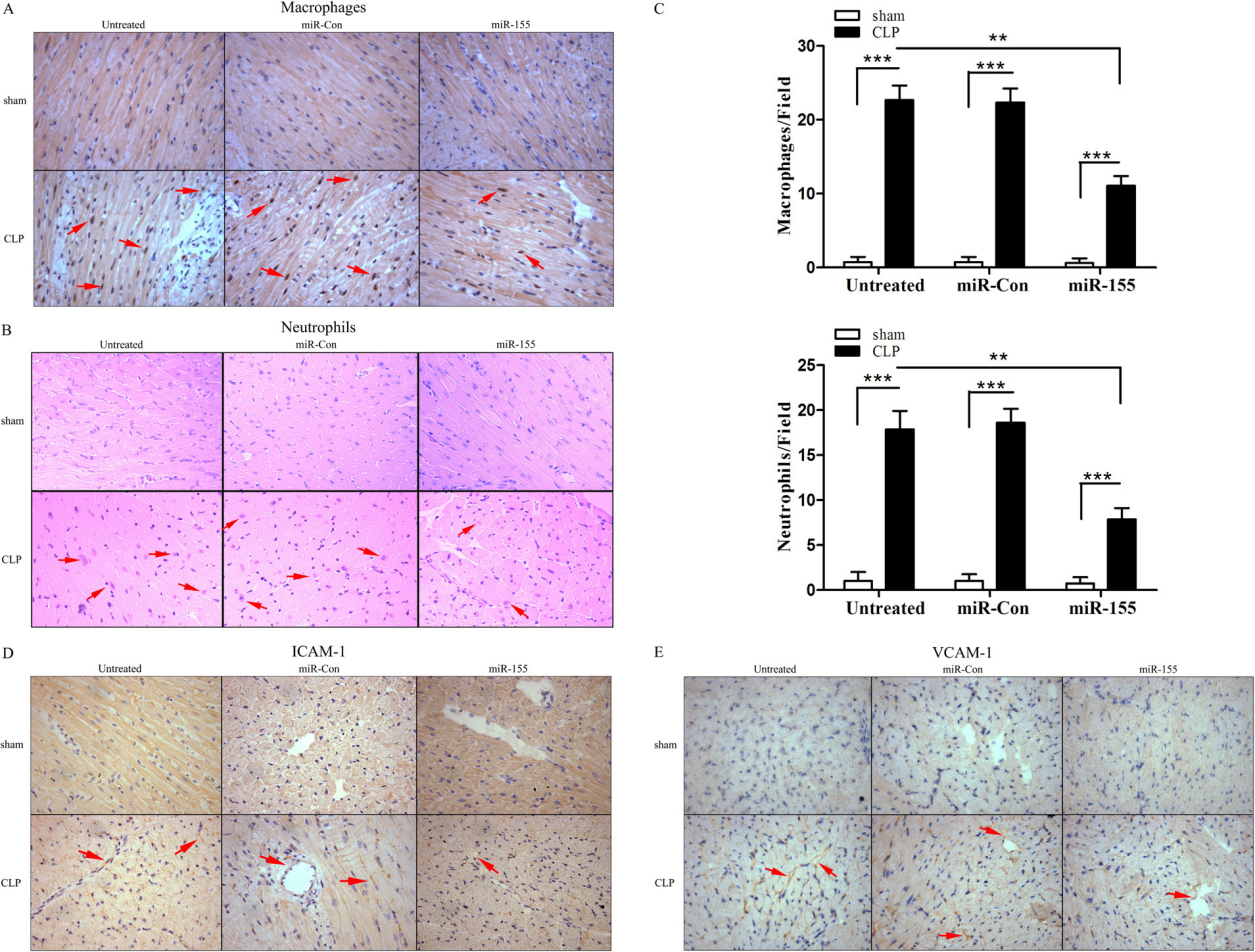
Transfection of miR-155 mimic prevents infiltration of macrophages and neutrophils into the myocardium and reduces adhesion molecule expression in late sepsis miR-155 mimic or miR-Con (80 mg/kg) was transfected through the tail vein 48 h after CLP. Hearts were harvested 12 days after CLP and sectioned for immunohistochemical staining to identify macrophages (**A**) and neutrophils (**B**). The bar graphs indicate the numbers of infiltrating macrophages and neutrophils in five heart fields (**C**). Transfection of miR-155 mimic attenuates late sepsis-induced ICAM-1 (**D**) and VCAM-1 (**E**) expression in the myocardium. There were five mice per group. **p < 0.01, ***p < 0.001 compared with indicated groups.

It has been reported that the infiltration of macrophages and neutrophils was markedly increased early into the myocardium following CLP, which contributes to cardiac dysfunction during the development of septic shock [19]. It remains unclear whether this phenomenon occurs in late sepsis. Figure 3A–3C shows that macrophages and neutrophils accumulation were markedly increased in the myocardium during the late-septic phase. miR-155 mimic transfection significantly reduced sepsis-induced infiltration of macrophages by 51.2% and neutrophils by 56% into the myocardium when compared with untreated CLP mice. It is well known that adhesion molecules, such as ICAM-1 and VCAM-1, play a critical role in inducing inflammatory cells infiltration into the tissues [19, 20]. Next, we examined the effect of miR-155 on the expression of adhesion molecules in the myocardium. As shown in Figure 3D and 3E, there is more positive staining of ICAM and VCAM-1 in the late-septic mice myocardium compared with sham control, and miR-155 mimic transfection attenuated sepsis-mediated expression of myocardial ICAM-1 and VCAM-1. CLP-induced accumulation of macrophages and neutrophils, or the expression of ICAM-1 or VCAM-1 in the myocardium was not affected when treated with miR-Con.

### Transfection of miR-155 mimic suppresses myocardium pro-inflammatory responses in late sepsis

**Figure 4:**
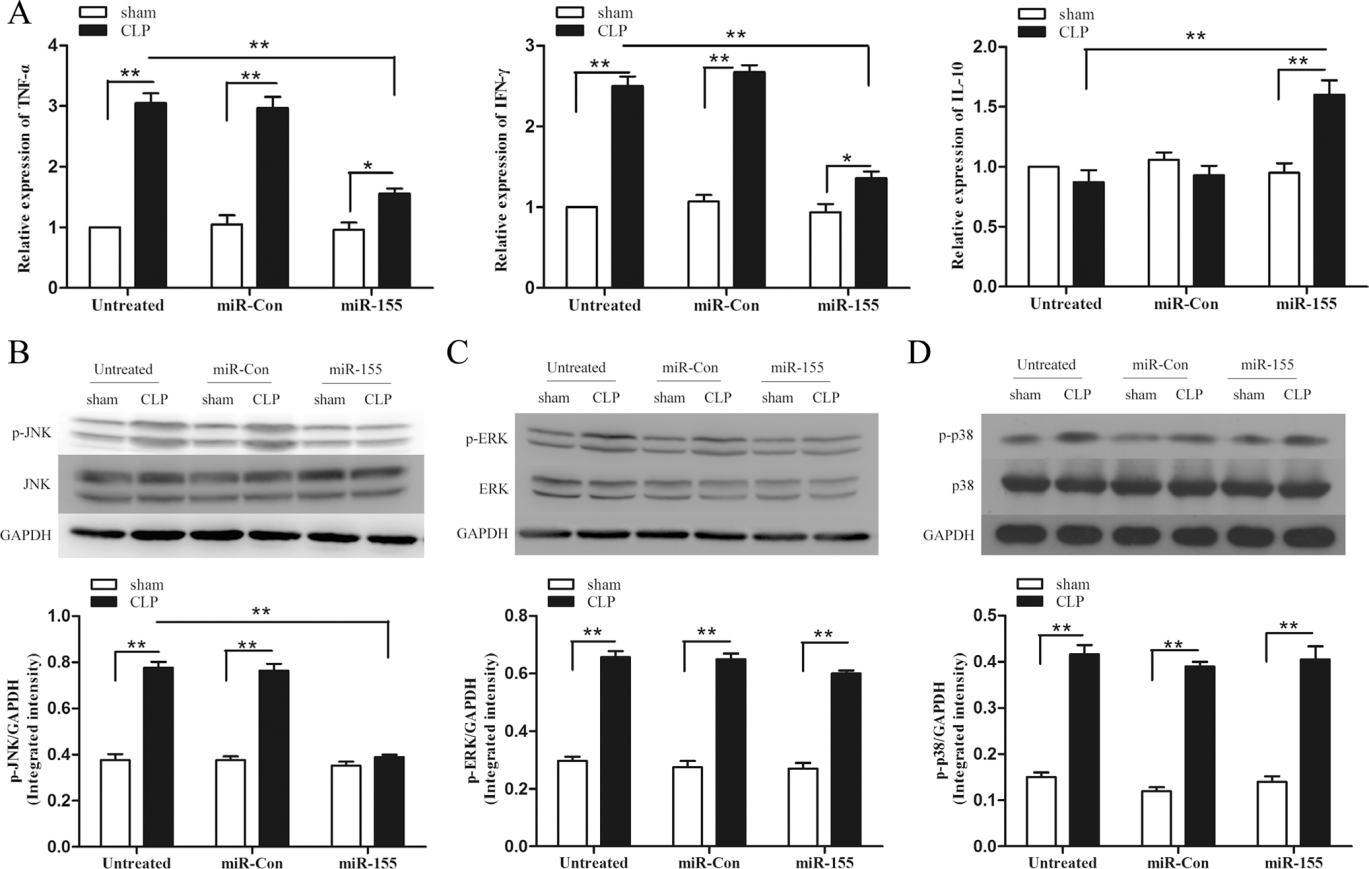
Transfection of miR-155 mimic attenuates inflammatory responses in late sepsis mice and suppresses p-JNK expression miR-155 mimic or miR-Con (80 mg/kg) was transfected through the tail vein 48 h after CLP. (**A**) Hearts were harvested for assay of cytokine TNF-α, IFN-γ and IL-10 by qPCR 12 days after CLP. (**B–D**) Levels of p38, phospho-p38, ERK, phospho-ERK, JNK and phospho-JNK in the myocardium were examined by immunoblotting with specific antibodies 12 days after CLP. GAPDH expression was used as an internal control. There were five mice per group. *p < 0.05, **p < 0.01 compared with indicated groups.

Increased infiltration of inflammatory cells into the myocardium and persistence of an inflammatory microenvironment contribute to cardiac dysfunction [21]. Then we examined the effect of increased expression of miR-155 on inflammatory cytokine production in late-septic mice myocardium. As shown in Figure 4A, CLP promoted significantly high expression of pro-inflammatory cytokine TNF-α (3-fold) and IFN-γ (2.5-fold) compared with sham control (set at 1-fold) on day 12 after sepsis. miR-155 mimic transfection reduced TNF-α, IFN-γ production by 48.9%, 45.6% respectively, and increased anti-inflammatory cytokine IL-10 (1.8-fold) in the myocardium compared with untreated CLP mice.

MAPK signaling pathway is an important component which regulates the expression of inflammatory cytokines and adhesion molecules [22]. To investigate the mechanisms responsible for the immunomodulation, we evaluated the role of miR-155 in activation of MAPKs. Figure 4B–4D shows that MAPKs were activated in the late-septic myocardium on day 12 after CLP. Interestingly, CLP-enhanced JNK phosphorylation in miR-155 mimic transfected heart was significantly decreased by 49.8% compared with untreated CLP mice. Whereas the dynamic of ERK1/2 and p38 activation was not altered after miR-155 mimic transfection. Our findings reveal in late sepsis, JNK is a downstream effector of miR-155. Transfection of miR-Con did not alter sepsis-induced MAPKs activity in the myocardium.

### Increased miR-155 inhibits cardiac Arrb2 expression in late sepsis

**Figure 5:**
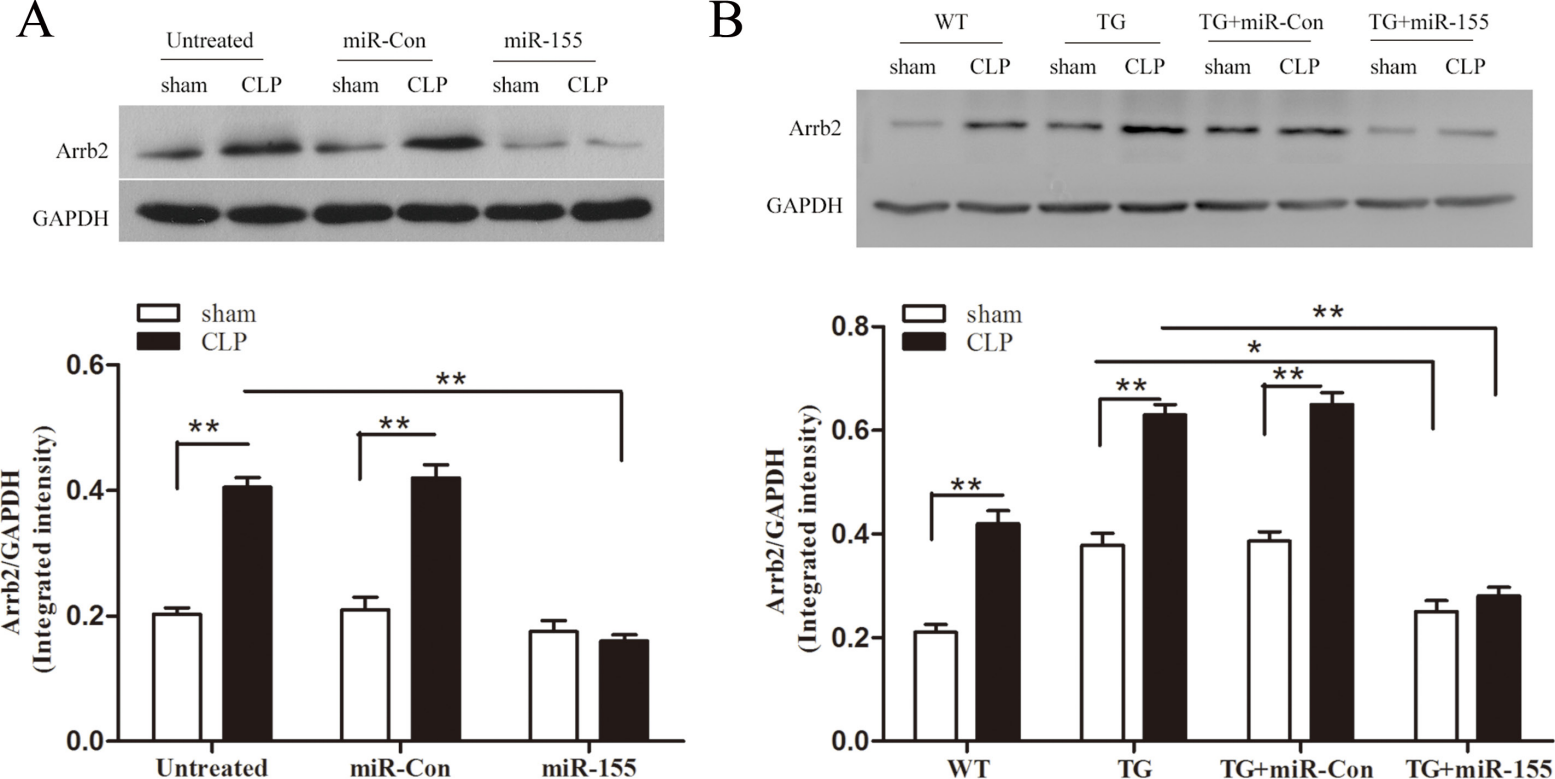
Transfection of miR-155 mimic inhibits myocardium Arrb2 expression in late sepsis miR-155 mimic or miR-Con (80 mg/kg) was injected through the tail vein 48 h following CLP. The level of Arrb2 expression in the myocardium was examined by immunoblotting in WT (**A**) and Arrb2 TG (**B**) mice 10 days following CLP. There were five mice per group. *p < 0.05, **p < 0.01 compared with indicated groups. WT, wild-type; Arrb2 TG, Arrb2 overexpression.

Using luciferase report assay, our previously research finds miR-155 could target Arrb2 and inhibit its expression *in vitro* [17]. In this study, we further explored the effect of miR-155 on Arrb2 expression in the late-septic myocardium. As shown in Figure 5A, CLP increased Arrb2 expression (2-fold) compared to sham control on day 10 after sepsis. Transfection of miR-155 mimic reduced the level of Arrb2 by 60.5% compared with untreated CLP mice. Using Arrb2 TG mice (Figure 5B), we detected the expression of Arrb2 in sham Arrb2 TG mice was 1.8-fold higher than in sham WT mice. miR-155 mimic transfection in Arrb2 TG mice decreased Arrb2 expression by 33.9% compared with sham Arrb2 TG mice. After CLP in Arrb2 TG mice, transfection of miR-155 mimic suppressed myocardial Arrb2 expression by 55.6% compared with untreated Arrb2 TG mice in late sepsis. Transfection of miR-Con did not alter the expression of Arrb2 in the presence and absence of sepsis.

### miR-155 improves Arrb2 TG mice survival and cardiac dysfunction in late sepsis

**Figure 6:**
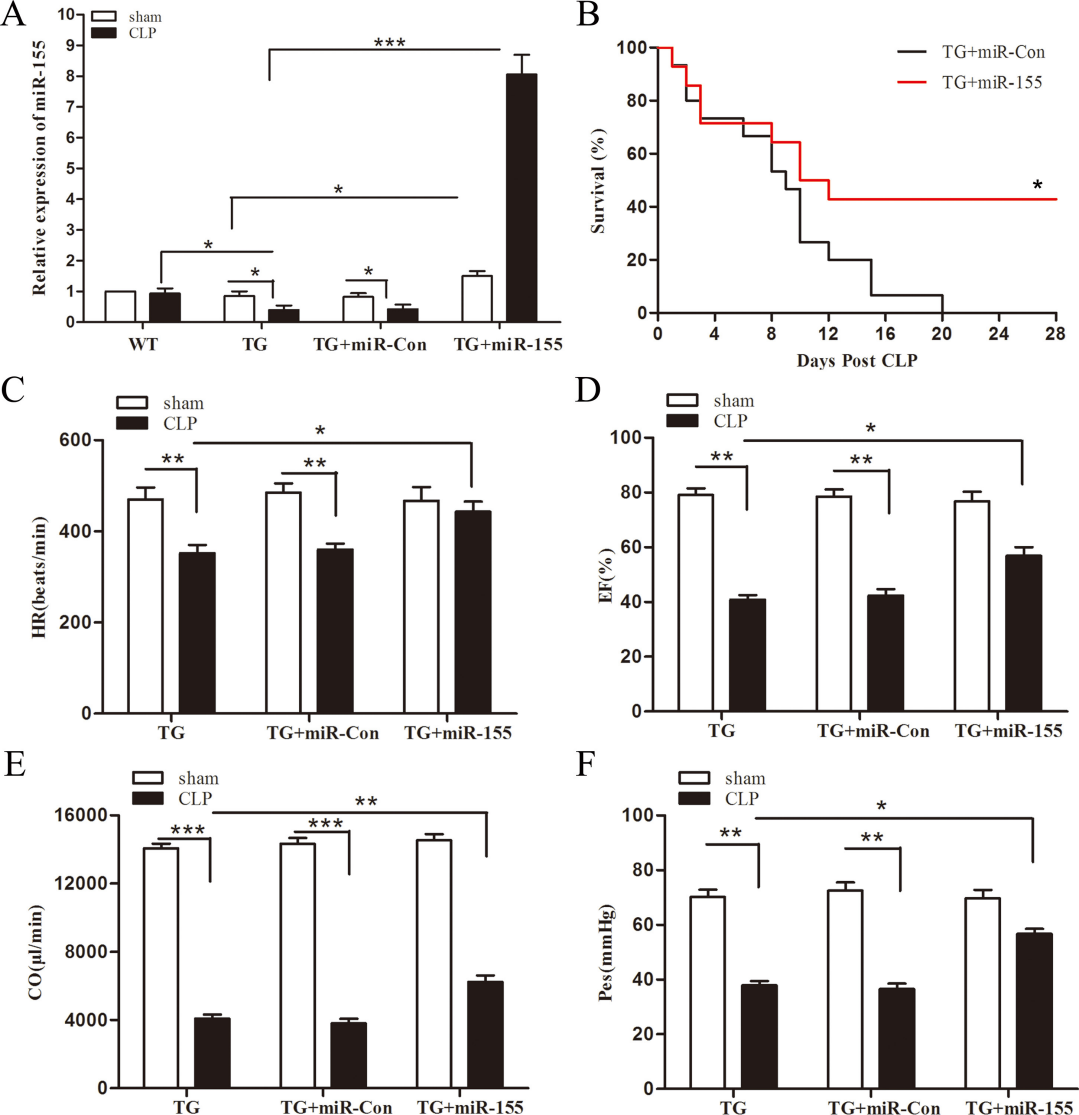
Increased expression of miR-155 attenuates Arrb2 overexpression (TG) mice cardiac dysfunction and improves survival in late sepsis Eight- to 10-weeks Arrb2 TG male mice were subjected to CLP or sham surgical operation. miR-155 mimic or miR-Con (80 mg/kg) was injected through the tail vein 48 h after CLP. (**A**) Hearts were harvested for assay of miR-155 by qPCR 10 days after CLP. There were four mice per group. (**B**) Arrb2 overexpression (TG) mice were transfected with miR-155 mimic or miR-Con 48 h after CLP and then monitored for survival for up to 28 days. There were 15-18/group. *p < 0.05 compared with Arrb2 TG + miR-Con group. Ten days after CLP, cardiac function measurement of Arrb2 TG mice was analysis by pressure-volume loop hemodynamic parameters. (**C**) HR, heart rate. (**D**) EF, ejection fraction. (**E**) CO, cardiac output. (**F**) Pes, left ventricular end-systolic pressure. There are five mice per group. *p < 0.05, **p < 0.01, ***p < 0.001 compared with indicated groups.

To further elucidate the relationship between miR-155 and Arrb2, and their effect on late-septic mice survival, we tested miR-155 expression in Arrb2 TG mice myocardium, meanwhile observed Arrb2 TG mice survival in late sepsis model. As shown in Figure 6A, miR-155 expression in CLP Arrb2 TG mice was reduced by 56.9% compared with in CLP WT mice, meaning that Arrb2 inhibits the expression of miR-155 and there may be a loop pathway between Arrb2 and miR-155. Transfection of miR-155 mimic increased miR-155 expression in the presence and absence of sepsis, when compared with untreated Arrb2 TG mice. Figure 6B indicated Arrb2 TG mice were susceptive to late sepsis. Death occurred with the highest frequency on day 6–10 after sepsis, and 100% mortality occurred at 20 days after CLP. Survival in miR-155 mimic transfection septic Arrb2 TG mice was improved by 40% compared with miR-Con group. We also evaluate the effect of miR-155 on cardiac function in Arrb2 TG mice at day 10 after sepsis. CLP induced significant cardiac dysfunction in Arrb2 TG mice by decreased heart rate (25.1%) (Figure 6C), EF% (48.3%) (Figure 6D), cardiac output (70.9%) (Figure 6E), and Pes (46%) (Figure 6F) compared with baseline values. miR-155 mimic transfection significantly attenuated sepsis-induced cardiac dysfunction, which increased heart rate by 25.9%, EF% by 39.1%, cardiac output by 52.4%, and Pes by 49.6% when compared with the untreated CLP TG group. Our results indicate that miR-155 may attenuate cardiac dysfunction through downregulation of the Arrb2 expression level.

### Increased miR-155 restores responsiveness of late-septic Arrb2 TG mice to LPS

**Figure 7:**
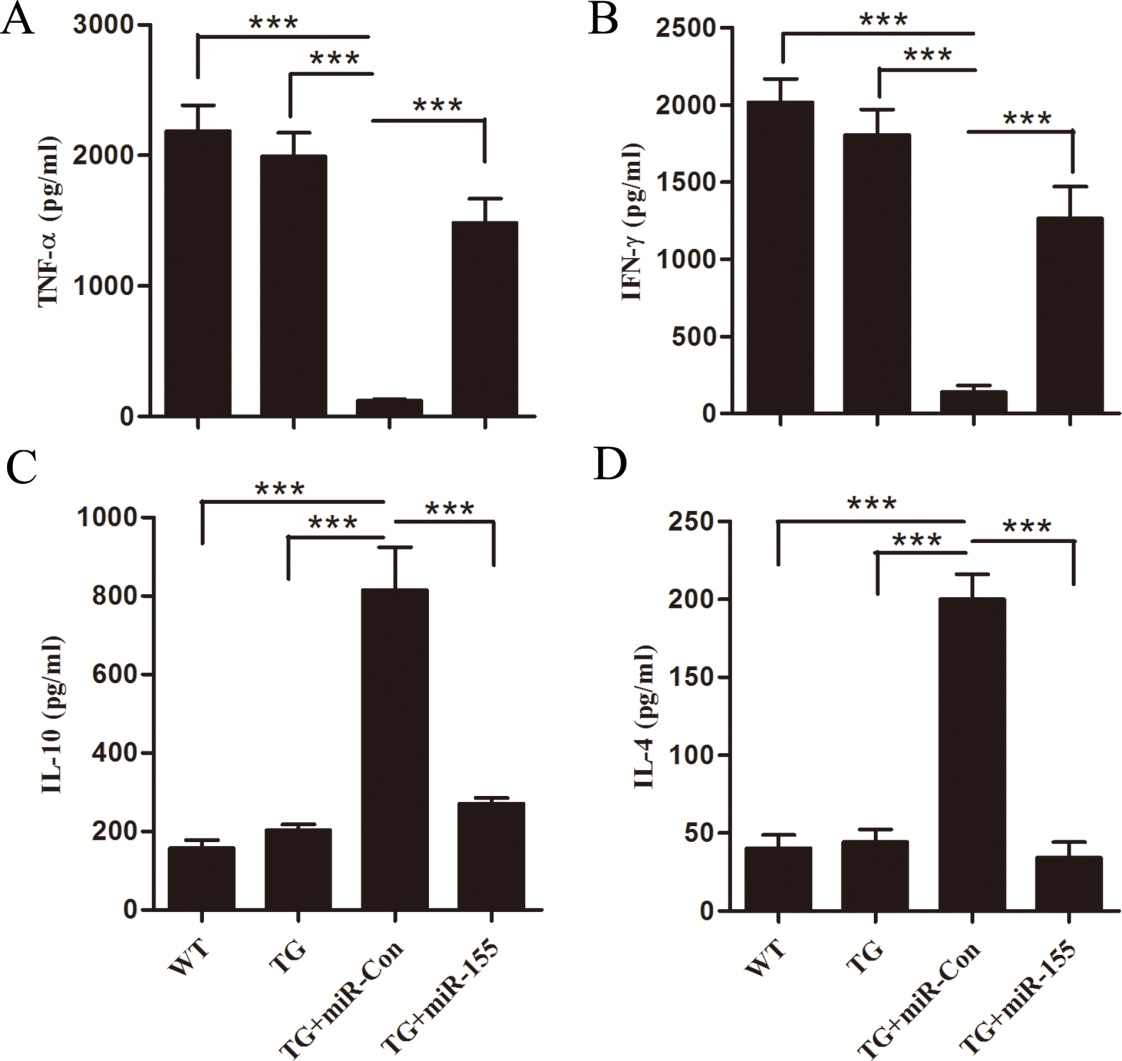
miR-155 mimic restores Arrb2 overexpression (TG) mice responsiveness to bacterial LPS in late sepsis At days 6 to 16 after CLP, mice were moribund (TG+miR-Con group) and a corresponding number of surviving, healthy-appearing mice (TG+miR-155 group) were challenged with 10 μg of bacterial LPS (i.p.). Sham-operated TG mice and wild-type mice also received LPS and served as positive controls. After 6 h, sera were obtained and cytokine levels of TNF-α, INF-γ, IL-10 and IL-4 were examined by ELISA. There are five mice per group. ***p < 0.001 compared with indicated groups.

Late-septic mice are shown in a state of immunosuppression, indicated by producing less pro-inflammatory but more anti-inflammatory cytokines in our previous study [23]. We hypothesized that mice which survived late sepsis due to miR-155 mimic transfection should be immunoreactive, and then they should resist to a challenge with secondary bacterial LPS. TG mice that survived (the miR-155 mimic group) or moribund (the miR-Con group) were pooled at days 6 to 16 after CLP and infected with LPS (10 μg, i.p). Sham-operated Arrb2 TG mice also received LPS and served as a positive control. The inflammatory response was determined by measuring the sera levels of the pro-inflammatory (TNF-α, INF-γ) and anti-inflammatory (IL-10, IL-4) cytokines 6 h after LPS challenge. As shown in Figure 7, the miR-155 mimic transfection in Arrb2 TG mice significantly increased TNF-α and INF-γ expression and reduced IL-10 and IL-4 production compared with miR-Con group. We also observed that the cytokine profiles in the miR-155 mimic transfection Arrb2 TG mouse were similar to those in sham-operated control group, suggesting that increased expression of miR-155 restored immunoreactivity to LPS, while Arrb2 TG mice transfection with miR-Con remained immunosuppressed.

## DISCUSSION

Sepsis is now the leading cause of death in the intensive care unit. Clinical observation finds that sepsis has biphasic death distribution, with an initial early peak at several days and a late peak at several weeks due to persistent organ injury or failure [24, 25]. However, the precise mechanisms of late sepsis mortality are still uncertain. Several studies suggest that immune dysfunction, immune suppression, and persistent catabolism and inflammation contribute to the deleterious state of chronic disease [26, 27]. Though some experimental agents, such as thymosin (NCT00711620) and granulocyte-macrophage colony stimulating factor (NCT00252915), are being tested in clinical trials to reconstitute immune responsiveness in patients with severe sepsis, an effective treatment is still unavailable until now. Using the late-septic mice model, our present study demonstrates that transfection of miR-155 mimic improves sepsis mice survival and attenuates inflammation-induced myocardium injury. We also found that Arrb2 exacerbated the mice mortality and immunosuppression in late sepsis. Increased expression of miR-155 inhibited cardiac Arrb2 expression, and then prevented Arrb2 TG mice cardiac dysfunction and restored late-septic Arrb2 TG mice immunoreactivity to LPS. Taken together, *in vivo* miR-155 mimic transfection attenuates septic sequelae and could pave the way toward new therapies.

The cardiovascular system plays an important role in the pathogenesis of sepsis. It has been shown that cardiac activation of PI3K/Akt-dependent signaling significantly attenuated myocardial dysfunction and improved survival in CLP-induced sepsis, which demonstrates a causal relationship between the protection of myocardial function and survival in early sepsis [28]. However, as recent researches suggest that benefits in early sepsis survival have been forfeited to escalating long-term sepsis mortality [25, 29], the changes of myocardial function during late-septic phase are still unclear. Here, we found CLP also induced significant cardiac dysfunction in late sepsis mice by decreased heart rate, cardiac output, EF%, and Pes. Therefore, clarifying the pathogenesis of late sepsis-induced myocardial dysfunction will provide benefits to seek specific therapy.

miRNAs are known to modulate normal physiological and pathophysiological signals within multiple organ systems [10, 11, 30]. We focused our attention on the expression of miR-155, which is closely related to a variety of cardiovascular diseases. miR-155 may be a potential prognostic indicator for heart failure [12]. miR-155 was found to repress eNOS expression, and regulate endothelium dependent vasorelaxation [31]. In our study, miR-155 level in the myocardium was increased during early sepsis and returned to baseline level in late sepsis. After transfection with miR-155 mimic, the expression of miR-155 was sustained increase during late-septic phase. Interestingly, miR-155 mimic treatment significantly attenuated CLP-induced cardiac dysfunction, and most importantly, increased miR-155 improved late-septic mice survival. A wide variety of stimuli, such as TLR ligands, can increase miR-155 expression, and then promotes both antimicrobial and antiviral responses of macrophages [32, 33]. miR-155 is required for the effective control of streptococcus pneumonia from the nasopharynx, and antipneumococcal adaptive immunity is broadly impaired in miR-155 knockout mice [34]. Mice with a deficiency of miR-155 are highly susceptible to herpes simplex encephalitis [35], suggesting that miR-155 can enhance the protective immune response to pathogens.

Endothelial cell activation is involved in septic injury in multiple organs [36]. Systolic cardiac dysfunction is directly associated with endothelial dysfunction in septic patients. Myocardium-depressing factors, such as TNF-α, can increase the expression of ICAM-1 and VCAM-1 in coronary endothelial cells and cardiomyocytes, and blockade of VCAM-1 or ICAM-1 abrogates LPS-induced cardiac dysfunction [37]. *In vivo* transfection of miR-155 mimic, we found miR-155 attenuated late sepsis-mediated expression of myocardial ICAM-1 and VCAM-1. Meanwhile, miR-155 significantly reduced sepsis-induced infiltration of macrophages and neutrophils into the myocardium. miR-155 is also one of the most widely studied miRNAs in regulating inflammation. We observed miR-155 mimic transfection decreased TNF-α, IFN-γ production and increased anti-inflammatory cytokine IL-10 in the myocardium, and then attenuates the inflammatory microenvironment which contributes to cardiac dysfunction.

MAPK pathways have been implicated in different aspects of cardiac regulation. The ERK1/2 pathway is found to be responsive mainly to stimulation of growth signaling, JNK and p38 are selectively responsive to pathophysiological stressors, such as infection and cytokines [15, 38]. Our study shows that levels of phospho-p38, phospho-ERK and phospho-JNK were all increased in late-septic myocardium. However, CLP-enhanced JNK activity in miR-155 mimic transfected heart was significantly decreased, whereas the dynamic of ERK1/2 and p38 activation was not altered. Our findings indicate JNK is a downstream effector of miR-155 in late sepsis. Constitutively activated JNK can result in different aspects of pathological remodeling and may contribute to systolic dysfunction and slowed conduction velocity in the heart [39], which is consistent with our detection results of cardiac function. Previous research shows that miR-155 downregulates JNK activity through reducing the expression of PAK1 in mouse CD4+ T cells [40]. However, the precise mechanisms of miR-155 in the regulation of JNK activity in late-septic myocardium need to be further clarified.

Arrb2, a multifunctional adapter protein, plays important roles in both regulations of immune activation and cardiac function [14–16]. Arrb2 interacts with the adaptor TRAF6 to negatively regulate Toll-like receptor signaling in innate immunity [41]. Arrb2 mediates the binding of Src homology-containing tyrosine phosphatases to KIR2DL1 to regulate the inhibitory signaling of NK cells, and Arrb2-deficient mice were less susceptible to cytomegalovirus infection compared with wild-type mice [42]. In our study, Arrb2 TG mice were observed to be more susceptive to late sepsis than WT mice, as death occurred with the highest frequency on day 6–10 after sepsis, which was earlier than in WT mice (day 10–12). Our previous research has shown that Arrb2 is a direct target by miR-155 [17], so we further test the hypothesis that *in vivo* transfection of miR-155 mimic may reverse the effect of Arrb2 overexpression. CLP increased Arrb2 expression during the late-septic phase, and miR-155 mimic transfection reduced the level of Arrb2 expression, and then improved TG mice survival rate. We also observed the changes of cardiac function in late-septic TG mice, and found miR-155 mimic transfection significantly attenuated sepsis-induced cardiac dysfunction. Taken together, miR-155 may protect cardiac dysfunction through downregulation of the expression of Arrb2.

Recent studies indicate most patients surviving the early initial hyperinflammatory phase enter a long-term immunosuppressive phase [7, 25, 26]. Deaths in this immunosuppressive phase are mainly due to the deficit in controlling the primary infection or the susceptibility of secondary infections [43]. In our present study, late-septic Arrb2 TG mice were challenged with secondary bacterial LPS. We found that sepsis Arrb2 TG mice were immunosuppressive, characterized by reduced pro-inflammatory (TNF-α, INF-γ) and increased anti-inflammatory (IL-10, IL-4) cytokines expression. Transfection of miR-155 mimic restored TG mice immunoreactivity to LPS. Interestingly, we also observed that increased expression of miR-155 in the myocardium attenuated pro-inflammatory cytokine levels in late sepsis. Wang and colleagues observed that pharmacological inhibition of miR-155 using antagomiR improved cardiac function and suppressed cardiac apoptosis induced by LPS in acute septic mice [44]. The distinct animal models with immunological differences (acute sepsis: hyperinflammatory and late sepsis: immunosuppressive) could explain these seemingly conflicting experimental results. These findings highlight the importance of fully elucidating any miRNA mechanisms in the right time and in the right cell before pursuing miRNA-based therapies, and biomarker-guided immunotherapy at the proper immune phase needs further exploration.

In summary, the data presented here demonstrated that miR-155 protects against late sepsis-induced cardiac dysfunction and improves survival by attenuation of inflammatory responses at least partially via suppressing JNK activation. Increased miR-155 also inhibits Arrb2 expression and restores Arrb2 TG mice immunocompetency. miR-155/Arrb2 may be an effective target in the management of sepsis and in the field of infectious disease, and our mice model also provides an important insight into particular mechanisms of sepsis that may have a comparable role in humans.

## MATERIALS AND METHODS

### Experimental animals

Wild-type (WT) C57BL/6 mice were obtained from Jackson Laboratory (Bar Harbor, ME). Arrb2 overexpression (TG) mice were reported previously [42]. Briefly, full-length human Arrb2 cDNA from brain cDNA/λphage library was cloned into pcDNA3 with HA tag under the control of a human cytomegalovirus promoter. Then the DNA constructions were injected into fertilized mice eggs on a C57BL/6 background. Real-time PCR was used to check the transgene mRNA expression in founder mice and their offspring. The primers used to identify transgenic mice were Arrb2, sense 5′-CAG CCA GGA CCA GAG GAC A-3′, antisense 5′-TGA TAA GCC GCA CAG AGT T-3′. There is no difference between physical activity, productivity and life span in WT and Arrb2 TG mice. 8–10-week old male mice were used in survival and cardiac function analysis. All mice were maintained in the Division of Laboratory Animal Resources at East Tennessee State University (ETSU), a facility accredited by the Association for the Assessment and Accreditation of Laboratory Animal Care International. The animal experimental protocols were approved by the ETSU Committee on Animal Care.

### Cecal ligation and puncture (CLP) late sepsis model

Polymicrobial late sepsis was induced by CLP as described previously by us [23]. Briefly, mice were anesthetized via 5.0% isoflurane inhalation in 100% O_2_ in a closed chamber and then maintained with 2.5% isoflurane during surgery. A small anterior abdominal incision was made, and the cecum was ligated 1 cm proximal to the terminal of cecum with 2–0 silk, and then was punctured twice with a 23-gauge needle. A small amount of feces content was extruded into the abdominal cavity. The abdomen was then closed in layers with 3-0 silk. Sham-operated mice were processed identically, except without ligation and puncture. Immediately following surgery, 1 ml 0.9% saline was administrated by intraperitoneal (i.p.) injection for fluid resuscitation. To create the late sepsis phenotype, mice were subcutaneously administered antibiotic (imipenem, 25 mg/kg body weight) or an equivalent volume of 0.9% saline at 8 and 16 h after CLP.

### Transfection and injection of miR-155 mimic

Both mirVana *in vivo* ready miR-155 mimic and negative control (miR-Con) (Ambion, Carlsbad, CA) were complexed with Invivofectamine 3.0 (Invitrogen) reagent according to the manufacturer's protocol and our previous study [30], and were injected via the tail vein of male C57BL/6 mice at the dose of 80 mg/kg in 100 μl volumes. Injection was performed 48 h after CLP to allow initiation of sepsis.

### Endotoxin challenge

Surviving septic Arrb2 TG mice that received miR-155 mimic or miR-Con between days 6 and 16 after CLP were challenged (i.p.) with 10 μg of Gram-negative bacterial LPS (*E. coli* serotype 0111: B4; Sigma-Aldrich, St. Louis, MO). Meanwhile, sham-operated Arrb2 TG mice and wild-type mice also received LPS and served as a positive control. All mice were sacrificed after 6 h. Blood was obtained via cardiac puncture and sera were collected for cytokines measurement.

### Cardiac functional analysis

Cardiac function was detected by the SPR-839 instrument (Millar Instruments, Houston, TX, USA), and heart rate (HR), ejection fraction (EF), left ventricular end- systolic pressure (Pes), cardiac output (CO) were calculated as described previously [45]. Briefly, each mouse was intubated with a 22-gauge soft catheter and ventilated with a rodent ventilator (Columbus Instruments International Corp., Columbus, OH, USA), using a tidal volume of 0.3–0.5 ml and a respiratory rate of 110–120 breaths/min. After left thoracotomy, a microtip pressure-volume catheter was inserted through a 25-gauge apical stab into the left ventricular to measure the cardiac function. The signals were continuously recorded using an ARIA pressure-volume conductance system (Millar Instruments) connected to a Powerlab/4SPA/D converter (AD Instruments, Mountain View, CA, USA). All pressure-volume loop data were analyzed with PVAN3.4 (Millar Instruments). After the functional analysis, the hearts were removed and perfused for 2 min to remove the remaining blood. Portions of the midventricle were fixed for immunological studies.

### Quantitative PCR assay of miRs

miRs were isolated from heart tissues using the mirVana™ miR isolation kit (Ambion) according to the manufacturer's protocol. miR-155 levels were quantified by quantitative PCR (qPCR) using specific primers TaqMan MicroRNA Assay (primer identification numbers: 002571 for mmu-miR-155 and 001973 for snRU6) and TaqMan Universal PCR Master Mix (Applied Biosystems) on a Bio-Rad PCR instrument. miR-155 level was calculated using the 2^-ΔΔCT^ cycle threshold method after normalization to the snRU6 as an internal control [30].

### Real-time quantitative RT-PCR

The real-time PCR detection technique was performed as described in our previous publications [30, 46]. Total RNA was isolated from mouse heart using an RNeasy Plus Mini Kit (QIAGEN Sciences, MD). One microgram of RNA from each sample was used for reverse transcription using a Reaction Ready™ first strand cDNA synthesis kit (SABiosciences, Frederick, MD). PCR was performed using real-time™ SYBR Green Fluorescein PCR Master Mix (SABiosciences). GAPDH expression was used as an internal control. The primer sequences used were as follows: TNF-α sense 5′-AGG CAC TCC CCC AAA AGA TG-3′, antisense 5′-TCA CCC CGA AGT TCA GTA GAC AGA-3′; IFN-γ sense 5′-AGG AAC TGG CAA AAG GAT GGT GAC-3′, antisense 5′-TGA CGC TTA TGT TGT TGC TGA TGG-3′; IL-10 sense 5′-TGC TAA CCG ACT CCT TAA TGC AGG AC-3′, antisense 5′-CCT TGA TTT CTG GGC CAT GCT TCT C-3′; GAPDH sense 5′-TGA CCA CAG TCC ATG CCA TC-3′, antisense 5′-GAT GGG GGT TAC ACA GGC AG-3′.

### Detection tissue invading neutrophils and macrophages

Neutrophil accumulation in the myocardium was stained with naphtol AS-D Chloroacetate Esterase (Sigma-Aldrich, St. Louis, MO) according to the manufacturer's protocol. Macrophages in heart tissues were examined with the specific Ab F4/80 (1:50 dilution; Santa Cruz Biotechnology, Santa Cruz, CA). Three slides from each block were evaluated and observed with bright-field microscopy. The results are expressed as the numbers of macrophages/field (×40 magnification).

### Immunohistochemistry

Heart tissues were immersion-fixed in 4% buffered paraformaldehyde, embedded in paraffin. ICH was performed with a Diaminobenzidine (DAB) Histochemistry kit (Molecular Probes, Invitrogen, CA) according to the manufacturer's protocol and our previous studies [14, 46]. Briefly, following antigen retrieval with 1mM EDTA (pH 8.0), deparaffinized sections were incubated in 1% Blocking Reagent solution for 1 h at 37°C and then stained with anti-ICAM-1 (1:50 dilution; Santa Cruz Biotechnology) and anti-VCAM-1 (1:50 dilution; Santa Cruz Biotechnology) respectively, overnight at 4°C. After washing, sections were incubated with biotinylated anti-mouse IgG (H+L) (Vector Laboratories, Burlingame, CA) and subsequently with HRP conjugate. Finally, the signal was shown with DAB substrate and counterstained with hematoxylin. Three different areas of each section were evaluated.

### Western blot analysis

Western blot was performed as described previously [15, 47]. Samples containing equal amounts of protein extracted from heart tissue lysis were loaded into 10–15% SDS-PAGE, and then transferred to a nitrocellulose membrane. Blocking the membrane for 1 h at room temperature with 3% BSA. The membrane was incubated overnight at 4°C with the primary antibody. The signals were detected with the ECL system (Amersham Biosciences) and quantified by scanning densitometry using a Bio-Image Analysis System (Bio-Rad). Anti-phospho-p38 MAPK, anti-p38 MAPK, anti-JNK, anti-phospho-JNK, anti-ERK, anti-phospho-ERK, anti-Arrb2 and GAPDH antibodies were obtained from Cell Signaling Technology (Beverly, MA).

### ELISA for cytokine assay

Blood was collected from the experimental and control mice to determine the serum level of cytokines as described by us [46]. Samples were allowed to clot for 2 h at room temperature before centrifugation for 20 min at 2000 g. Then serum was removed and stored at –80°C for subsequent ELISA assay. The quantity of cytokines (IFN-γ, TNF-α, IL-4 and IL-10) was examined using a Quantikine Mouse ELISA kit (R&D Systems, MN).

### Statistical analysis

All quantitative data were presented as mean ± SD. Statistical analysis of data between groups was performed with two-tailed Student *t* test or one-way ANOVA for experiments with more than two subgroups. Survival curves were generated by the Kaplan-Meier method, and the log-rank test was used to compare group survival. *p* values < 0.05 were considered to be statistically significant.

## Abbreviations

CLP: cecal ligation and puncture
Arrb2: β-arrestin 2
miR: microRNA
MI: myocardial infarction
WT: Wild-type
Arrb2 TG: Arrb2 overexpression
i.p.: intraperitoneal
HR: heart rate
EF: ejection fraction
Pes: left ventricular end-systolic pressure
CO: cardiac output
qPCR: quantitative PCR
DAB: Diaminobenzidine.
